# The Critical Role of Early Dengue Surveillance and Limitations of Clinical Reporting – Implications for Non-Endemic Countries

**DOI:** 10.1371/journal.pone.0160230

**Published:** 2016-08-08

**Authors:** Jui-Hung Kao, Chaur-Dong Chen, Zheng-Rong Tiger Li, Ta-Chien Chan, Tsung-Hwa Tung, Yin-Hsia Chu, Hau-Yuan Cheng, Jien-Wei Liu, Fuh-Yuan Shih, Pei-Yun Shu, Chien-Chou Lin, Wu-Hsiung Tsai, Chia-Chi Ku, Chi-Kung Ho, Chwan-Chuen King

**Affiliations:** 1 Department of Medicine (Med.), College of Med., National Taiwan University (NTU), Taipei, Taiwan (100), Republic of China (R.O.C.); 2 Department of Health, Kaohsiung City Government, Kaohsiung, Taiwan (802), R.O.C.; 3 Institute of Epidemiology and Preventive Med., College of Public Health, NTU, Taipei, Taiwan (100), R.O.C.; 4 Research Center for Humanities & Social Science, Academia Sinica, Taipei, Taiwan (115), R.O.C.; 5 Department of Pediatrics, NTU Hospital, Taipei, Taiwan (100), R.O.C.; 6 Division of Infectious Diseases, Department of Internal Medicine, Kaohsiung Chang Gung Memorial Hospital, Chang Gung University Medical College, Kaohsiung, Taiwan (833), R.O.C.; 7 Department of Emergency Medicine, NTU Hospital, Taipei, Taiwan (100), R.O.C.; 8 Department of Health, Taiwan Centers for Disease Control (Taiwan CDC), Taipei, Taiwan (100), R.O.C.; 9 Department of Public Health, Kaohsiung Medical University, and Dept. of Occupational and Environmental Med., Kaohsiung Medical University Hospital, Kaohsiung, Taiwan (807), R.O.C.; 10 Institute of Immunology, College of Medicine, NTU, Taipei, Taiwan (100), R.O.C.; Curtin University, AUSTRALIA

## Abstract

The increasing dengue burden and epidemic severity worldwide have highlighted the need to improve surveillance. In non-endemic areas such as Taiwan, where outbreaks start mostly with imported cases from Southeast Asia, a closer examination of surveillance dynamics to detect cases early is necessary. To evaluate problems with dengue surveillance and investigate the involvement of different factors at various epidemic stages, we investigated 632 laboratory-confirmed indigenous dengue cases in Kaohsiung City, Taiwan during 2009–2010. The estimated sensitivity of clinical surveillance was 82.4% (521/632). Initially, the modified serological surveillance (targeting only the contacts of laboratory-confirmed dengue cases) identified clinically unrecognized afebrile cases in younger patients who visited private clinics and accounted for 30.4% (35/115) of the early-stage cases. Multivariate regression indicated that hospital/medical center visits [Adjusted Odds Ratio (aOR): 11.6, 95% confidence interval (CI): 6.3–21.4], middle epidemic stage [aOR: 2.4 (1.2–4.7)], fever [aOR: 2.3 (2.3–12.9)], and musculo-articular pain [aOR: 1.9 (1.05–3.3)] were significantly associated with clinical reporting. However, cases with pruritus/rash [aOR: 0.47 (0.26–0.83)] and diarrhea [aOR: 0.47 (0.26–0.85)] were underreported. In conclusion, multiple factors contributed to dengue surveillance problems. To prevent a large-scale epidemic and minimize severe dengue cases, there is a need for integrated surveillance incorporating entomological, clinical, serological, and virological surveillance systems to detect early cases, followed by immediate prevention and control measures and continuous evaluation to ensure effectiveness. This effort will be particularly important for an arbovirus, such as Zika virus, with a high asymptomatic infection ratio. For dengue- non-endemic countries, we recommend serological surveillance be implemented in areas with high *Aedes* mosquito indices or many breeding sites. Syndromic surveillance, spatial analysis and monitoring changes in epidemiological characteristics using a geographical information system, as well as epidemic prediction models involving epidemiological, meteorological and environmental variables will be helpful for early risk communication to increase awareness.

## Introduction

In recent decades, the global incidence of dengue virus (DENV) infection has increased dramatically, with epidemics involving more severe cases [1, 2]. In non-endemic countries, outbreaks have typically started from imported cases [3], so adequate surveillance to detect early cases is crucial. As indigenous dengue cases increase domestically, surveillance and public health efforts must consider changes in epidemiologic characteristics across different stages of an epidemic process. In Taiwan, dengue cases have occurred annually since 1987 [4], and outbreaks have mostly begun with imported cases from Southeast Asian dengue-endemic areas with high incidence of dengue hemorrhagic fever (DHF) or severe dengue cases [5]. Taiwan’s case is thus similar to those in many European countries [3], the US [6], and Japan [7]. Reporting of every clinically probable dengue case (whether mild or severe) is mandatory in Taiwan (i.e. passive surveillance), which differs from surveillance approaches in most dengue-endemic Asian countries, where only DHF cases must be reported [8]. Although most laboratory-confirmed dengue cases in Taiwan have been detected through passive surveillance (physicians’ clinical reporting), serological surveillance to search for possible cases with an epidemiologic link to the confirmed ones (see the METHODS) has proven effective in quickly identifying unreported dengue cases [9, 10]. As epidemiological characteristics change from the early to middle and late stages of an epidemic, the focus of efforts in prevention and control measures should vary in strategy across stages. Therefore, it is crucial to modify dengue surveillance at different epidemic stages, to identify cases early, speed up subsequent public health interventions and stop further viral transmission.

Serological surveillance for early detection of initial cases and immediate mosquito control have resulted in containment of indigenous dengue epidemics in southern Taiwan [9, 10], as reflected by the few sporadic dengue cases occurring before and during the World Games in a large city—Kaohsiung during July 16–27, 2009. This involved spending approximately 111 million US dollars by local and central government agencies on enhanced mosquito surveillance, routine cleaning and inspection of breeding sites surrounding game areas. Additionally, ovitraps were set up at 22 game sites starting on June 1, 2009. However, an unexpected increase in dengue cases occurred in Kaohsiung in September 2009. Therefore, the effectiveness of the surveillance used in this outbreak needs to be reexamined. The specific aims of this study were to: (1) estimate the sensitivity of clinical surveillance and evaluate any problems in the surveillance system, (2) investigate factors involved in missed and delayed reporting, (3) integrate clinical and epidemiological characteristics (e.g. age groups, medical visit behaviors, and clinical manifestations) to improve surveillance, and (4) monitor the whole epidemic process from early through late stages to better assess public health implications. In dengue-endemic countries, multiple DENV serotypes have co-circulated. However, dengue epidemics in Taiwan before and during 2009–2010 as well as other non-endemic countries were most likely attributable to one predominant DENV serotype. Moreover, dengue cases occur annually with different serotypes, so these countries have yet to reach endemicity. In such areas, it is better to monitor clinical and epidemiological changes of dengue cases at different epidemic stages, as done in this study. While global warming has expanded the geographical distribution of dengue cases [11–13], we believe our experiences may help minimize the threat of dengue in Europe, the Americas, and other non-endemic areas.

## Materials and Methods

### Study Areas

Kaohsiung City [population of 1,527,914 persons (as of December 2009) covering 153.6 square kilometers] [14] (S1 Fig), is a metropolis in southern Taiwan. It has a tropical climate with monthly mean temperatures ranging from 19.3 to 29.2°C, suitable for both *Aedes aegypti* and *Aedes albopictus* mosquitoes to transmit dengue viruses [15]. Dengue epidemics (caused by different predominant DENV serotypes) occurred in this city almost every year from 1987 to 2009; except in case of warm winters or failed efforts at control, when viral transmission of the same serotype occurred continuously across two different years [5, 16–18]. Most of these epidemics started when two conditions were met: appropriate meteorological factors for *Aedes* [15], and imported cases from dengue-endemic Southeast Asian countries [5]. In fact, Kaohsiung’s dengue cases accounted for 26.8% (2087/7793) [ranging from 7.4% to 60.4%] of Taiwan’s total dengue cases from January 2003 to December 2010 [14]. Therefore, we used the large (epidemic-scale) numbers of dengue cases in Kaohsiung during 2009–2010 to investigate possible factors affecting dengue reporting.

### Surveillance, Study Populations and Definition of Dengue Cases

**Fig 1:**
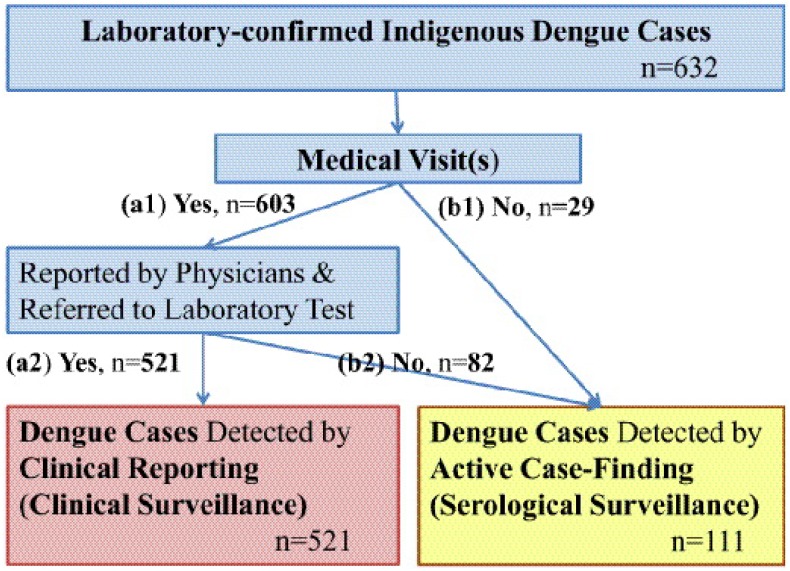
Laboratory confirmed indigenous dengue cases in Kaohsiung, Taiwan detected through the two surveillance systems in Kaohsiung Department of Health and Taiwan-CDC, from 11 July 2009 to 13 February 2010. The two surveillance systems include clinical and serological surveillance systems. Clinically unreported cases or patients who had not visited medical care may be picked up by a case-finding approach through epidemiological investigation and serological surveillance. In the past, additional blood samples of neighbors, work colleagues, and staff and students at schools must be taken by local public health personnel as part of epidemiological investigation for dengue tests through serological surveillance, once a dengue case is laboratory confirmed. However, the serological surveillance effort in Kaohsiung from 2009 through 2010 collected only family members of confirmed cases who voluntarily gave blood samples but not all neighbors, work colleagues, and school classmates and teachers with an epidemiological link with the confirmed cases due to budget cut.

Surveillance of dengue cases in Taiwan consists of two parts—passive and active (Fig 1). In passive surveillance, physicians report clinically suspected cases and patients voluntarily self-report dengue cases to the health administration within 24 hours. Active surveillance measures such as hospital-based fever surveillance during outbreaks, and airport fever screening [19] have also detected cases. Additionally, a “responsive case-finding policy” to conduct epidemiological investigations and serological tests is routinely implemented once a dengue case is laboratory confirmed [9, 10]. Local health officials must initiate a field epidemiological investigation by (1) searching for additional dengue-like cases in a 50- and 100-meter radius (for a single case or cluster, respectively) surrounding each confirmed dengue case’s living, working, visiting or schooling areas, (2) monitoring close contacts of confirmed cases, and (3) collecting blood specimens for diagnosis, although only (2) and (3) have been in effect since 2009 due to governmental budget cuts.

### Case Definition and Viral Diagnosis

A “suspected dengue case” was defined as a patient with clinical symptoms compatible with dengue-like illness, dengue fever, or severe dengue symptoms with an epidemiologic link to a laboratory confirmed dengue case or with a travel history to dengue outbreak/endemic areas. A “probable dengue case” was defined as a patient with acute onset of fever (ear temperature > 38°C) plus at least two of the following symptoms/signs: headache, retro-orbital pain, myalgia, arthralgia, rash, hemorrhagic manifestations, and leukopenia. DHF cases were defined according to WHO criteria [20]. A “laboratory confirmed DENV infected case” is defined as one with single acute or paired (acute and convalescent) blood samples of suspected or probable dengue cases showing any positive laboratory results by viral isolation, anti-DENV-IgM, 4-fold serotiter rise in DENV-IgG antibodies in paired serum samples, DENV-RNA by reverse-transcription polymerase chain reaction (RT-PCR), or DENV-NS1 antigen as previously described [21, 22]. Serotypes were based mostly on RT-PCR results. As DENV-3 was the predominant serotype found in indigenous cases in this study, we excluded one indigenous DENV-2 case, five DENV-RNA-positive imported cases (1 DENV-1, 2 DENV-2 and 2 DENV-3) detected through airport fever surveillance, and patients with only dengue-IgG-positive results (negative for dengue-IgM, dengue-NS1 antigen, and dengue-RNA by RT-PCR), which may be related to past infection.

### Data Collection

All laboratory confirmed indigenous and imported dengue cases in this study came from passive reporting through clinical surveillance and case-finding DENV-infected persons through epidemiological investigation and serological surveillance in Kaohsiung from July 11, 2009 to February 13, 2010 documented by the Kaohsiung City’s Department of Health (KH-CDH) and the Centers for Disease Control in Taiwan, Republic of China (Taiwan-CDC). Weekly numbers of reported suspected dengue cases from the KH-CDH were also collected for better comparison.

Clinical, epidemiological and demographical data for each confirmed dengue case were recorded by local field epidemiologists and clinicians when the case was reported to the KH-CDH. These included: (1) clinical symptoms and signs (fever, headache, retro-orbital pain, arthralgia, myalgia, dry mouth, anorexia, nausea, vomiting, diarrhea, rash, itching, and hemorrhage), and (2) epidemiological and demographic information (age, gender, residential district, history of physician consultation due to dengue illness, and laboratory results). To evaluate reporting efficiency, three documented dates, including dengue illness onset dates, notification dates to the health administration (by physician or sero-surveillance), and hospital/clinic visit dates, were used to identify factors possibly associated with clinical reporting delay. Two terms are used here: (1) “reporting delay” is the number of days between the dengue illness onset date and the case’s reporting date through clinical surveillance system, and (2) “detection-delay days” is the numbers of days between the dengue illness onset date and the date that the case was identified through active case-finding by public health professionals.

### Data Analyses and Statistical Tests

**Fig 2:**
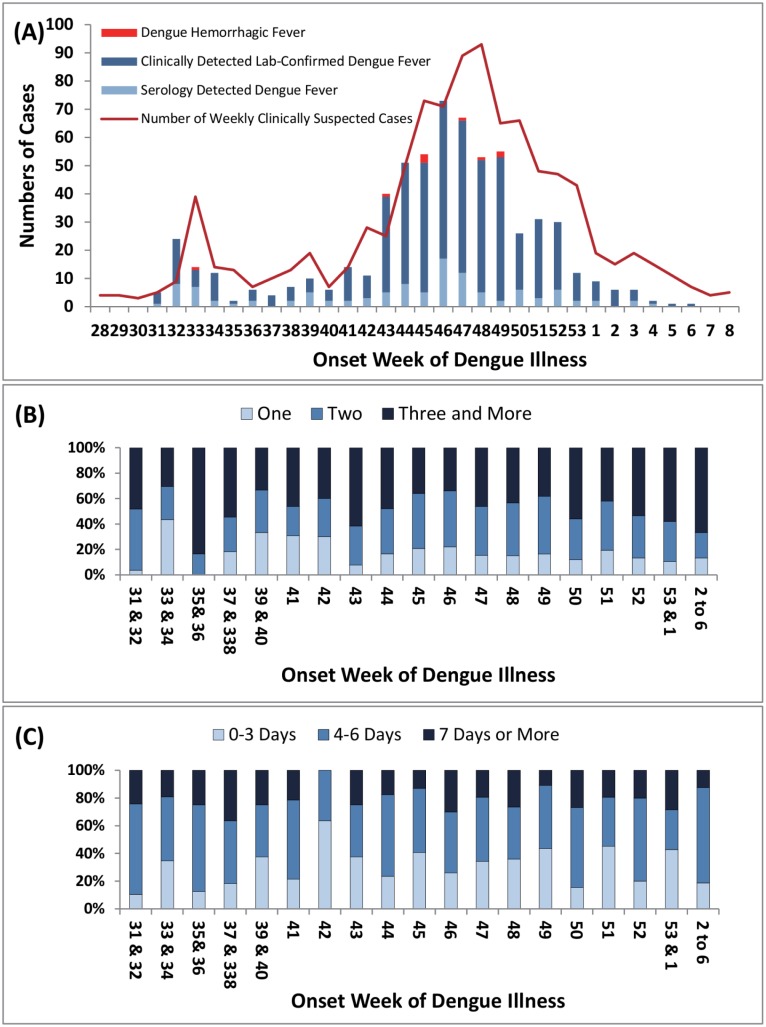
**(A) Weekly numbers of confirmed indigenous dengue cases:** Dengue cases (dengue fever and dengue hemorrhagic fever cases) were identified through both clinical and serological surveillance systems in Kaohsiung City (from 28th week, 2009 to 7th week, 2010). Epidemic curve shows case numbers over time for dengue fever and dengue hemorrhagic fever cases detected by clinical and serological surveillance systems. **(B) The weekly numbers of medical visits before notification to Kaohsiung DOH.** Darker blue represents dengue patients in this epidemic seeking more medical visits (i.e. hospital shopping). **(C) Weekly “Detection-delay Days” through the two surveillance systems.** Darker blue color indicates longer detection-delay through clinical and serological surveillance systems. The means and medians of “detection delay days” around five days at the three epidemic stages showing differences in distributions but without statistical differences [**means:** early = 5.3±3.4, middle = 5.0±3.9, late = 5.1±3.3, p = 0.71; **media**: 5, 4, 5, p = 0.48; **ranges:** 0–27, 0–47, and 0–24]. In (B) and (C): Those weeks with low numbers were summarized to better understand the trends.

Weekly numbers of confirmed dengue cases were summated according to illness onset dates, and are displayed in Fig 2A according to their sources of surveillance systems. To investigate possible changes in reporting behaviors with respect to different epidemic stages, we further divided this epidemic into “early stage”, “middle stage”, and “late stage” defined as before the 42nd week, 43rd to 49th week, and after the 50th week, respectively. This was based on data showing an approximate 4-fold increase in dengue case numbers from the 42nd to 43rd week and a striking halving of cases between the 49th and 50th week (Fig 2A).

Cases reported by physicians and those initially unreported through clinical surveillance but later identified through case-finding were compared by clinical (symptoms/signs and days after onset of dengue illness), epidemiological (epidemic stage), and demographical (age and gender) characteristics (Tables 1 and 2). Correlations between symptoms were systematically examined; then interrelated symptoms (e.g. myalgia/arthralgia, rash/itching, and anorexia/nausea/vomiting were grouped for analyses (Tables 3 and 4). Differences in clinical reporting regarding hospital levels, age, frequency of hospital/clinic visits, and symptoms/signs in the three stages were further analyzed. A chi-square test and t-test were conducted for categorical and continuous data, respectively, with p < 0.05 considered statistically significant.

**Table 1:** Comparison between reported versus unreported indigenous laboratory confirmed dengue cases in surveillance, epidemiology and clinical symptoms after medical visits in Kaohsiung City, during 2009–2010 epidemic of dengue/DHF.

Variable	Reported	Unreported	p-value
	(n = 521)(%)	(n = 82)(%)	
**A. Surveillance**			
**Last Med Visit Before Notification**			
**Local clinics**	6.1	**43.9**	**<0.01**
**Dist./Reg. Hosp.**	73.3	40.2	
**Med Centers**	20.5	15.9	
**No of Med Visits Before Notification**			
**1**	15.4	**30.5**	**<0.01**
**2**	37.4	37.8	
**≧3**	47.2	31.7	
**Detection-delay-days**			
**0–3**	35.1	17.1	**<0.01**
**4~6**	50.7	26.8	
**7+**	14.2	**56.1**	
**Mean ± SD**	4.4±2.1	8.7±7.2	**<0.01**
**B. Epidemiology**			
**Epidemic Stages**			
**Early**	15.4	**26.8**	**0.02**
**Middle**	65.1	51.2	
**Late**	19.6	22.0	
**Age (yrs)**			
**0~25**	17.3	**26.8**	**0.02**
**26~55**	48.2	52.5	
**56+**	34.5	20.7	
**C. Clinical Symptoms/Signs**			
**Fever**	96.2	82.9	**<0.01**
**Rash**	48.8	64.6	**<0.01**
**Itching**	19.6	40.2	**<0.01**
**Diarrhea**	24.0	34.1	**0.0495**
Headache	54.1	51.2	0.62
Myalgia	53.7	47.6	0.30
Arthralgia	53.9	43.9	0.09
Dry Mouth	38.0	41.5	0.55
Nausea	25.0	20.7	0.41
Vomiting	16.1	14.6	0.73
Retroorbital Pain	9.8	14.6	0.18
Hemorrhage	3.8	6.1	0.37

**Table 2:** Comparison between reported versus unreported indigenous laboratory confirmed dengue cases in surveillance, epidemiological characteristics and clinical symptoms, through the three epidemic stages in Kaohsiung City, during 2009–2010 epidemic of dengue/DHF.

	Early Stage			Middle Stage			Late Stage		
	Reported	Unreported		Reported	Unreported		Reported	Unreported	
	(N = 80)(%)	(n = 22)(%)		(n = 339)(%)	(n = 42)(%)		(N = 102)(%)	(N = 18)(%)	
**A. Surveillance**									
**Last Med Visit before Notification**									
** Local Clinics**	2.5	45.5	**<0.01**	6.5	40.5	**<0.01**	7.8	50.0	**<0.01**
** Dist./Reg.Hosp.**	76.3	45.5		76.4	40.5		60.8	33.3	
** Med Centers**	21.3	9.1		17.1	19.0		**31.4**	16.7	
**No. Med. Visits**									
** 1**	16.3	50.0	**0.01**	15.9	23.8	0.30	12.7	22.2	0.43
** 2**	30.0	40.9		40.1	42.9		34.3	22.2	
** ≧3**	53.8	9.1		44.0	33.3		52.9	55.6	
**Detection-delay (days)**									
** 0–3**	27.5	31.8	0.17	37.8	11.9	**<0.01**	32.4	11.1	**0.04**
** 4~6**	56.3	36.4		48.7	19.0		52.9	33.3	
** 7+**	16.3	31.8		13.6	**69.0**		14.7	**55.6**	
**B. Epidemiology**									
**Age (yrs)**									
** 0~25**	18.7	**45.5**	**0.02**	16.2	21.4	0.44	19.6	16.6	0.39
** 26~55**	56.3	45.5		45.7	50.0		50.0	66.7	
** 56+**	25.0	9.0		38.1	28.6		30.4	16.7	
**C. Clinical Symptoms/Signs**									
**Fever**	98.8	77.3	**<0.01**	95.3	90.5	0.19	97.1	72.2	**<0.01**
**Rash**	47.5	72.7	**0.04**	45.7	61.9	**0.048**	59.8	61.1	0.92
**Itching**	27.5	50.0	**0.046**	17.1	40.5	**<0.01**	21.6	27.8	0.56
**Diarrhea**	30.0	36.4	0.57	22.1	35.7	**0.051**	25.5	27.8	0.84

**Table 3:** Multivariate analyses on clinically reported and confirmed indigenous dengue cases, using a stepwise logistic regression full-model considering potential covariates and the three-stage-specific (early-, middle-, and late-stage) models.

Variable	Full Model		Early-Stage-Specific Model		Middle-Stage-Specific Model		Late-Stage-Specific Model	
	Adjusted OR (95% CI)	P-value	Adjusted OR (95% CI)	P-value	Adjusted OR (95% CI)	P-value	Adjusted OR (95% CI)	P-value
**Visited Hosp./Med. Center**	11.57 (6.26–21.38)	**<0.001**	28.29 (3.93–203.48)	**<0.001**	8.89 (4.13–19.10)	**<0.001**	9.25 (2.68–31.95)	**<0.001**
**Fever**	4.84 (2.04–11.47)	**<0.001**	18.89 (1.40–254.13)	**0.027**			7.98 (1.40–45.67)	**<0.020**
**Rash/Itching** ^a^	0.47 (0.26–0.83)	**0.010**	0.21 (0.05–0.95)	**0.043**	0.42 (0.20–0.87)	**0.020**		
**Middle Stage** ^b^	2.40 (1.24–4.65)	**0.009**						
**Myalgia/ Arthralgia** ^a^	1.85 (1.05–3.27)	**0.034**						
**Diarrhea**	0.47 (0.26–0.85)	**0.013**						
**Frequency of Med Visit**			3.523	**0.004**				
**AIC** ^c^	387.389		67.990		232.615		85.159	

^a^ Clinical presentations were combined to one variable by organ system: cutaneous (rash or Itching), musculoskeletal (myalgia or arthralgia), and upper gastrointestinal (anorexia, nausea or vomiting).

^b^ Stage1 as the reference.

^c^ Forward stepwise selection was conducted to select the model with minimal Akaike Information Criterion (AIC)

**Table 4:** Multivariate analyses on clinically reported and confirmed indigenous dengue cases, using stepwise logistic regression analyses to establish three age-group-specific models.

Variable	Lower-Age-Group-Specific Model (≤25 Years Old)		Middle-Age-Group-Specific Model (26–55 Years Old)		Higher-Age-Group-Specific Model (≥56 Years Old)	
	Adjusted OR (95% CI)	P-value	Adjusted OR (95% CI)	P-value	Adjusted OR (95% CI)	P-value
**Visited Hosp./ Med. Center**	22.34 (5.82–85.76)	**<0.001**	7.18 (3.03–17.03)	**<0.001**	26.88 (5.43–133.10)	**<0.001**
**Middle Stage** ^a^	6.63 (1.50–29.25)	**0.013**				
**Myalgia/ Arthralgia**	6.57 (1.68–25.62)	**0.007**				
**Rash/ Itching**			0.388 (0.18–0.86)	**0.019**		
**Diarrhea**			0.36 (0.17–0.86)	**0.008**		
**Anorexia/Nausea/ Vomiting**					3.67 (1.04–12.99)	**0.044**
**AIC** ^b^	76.313		213.324		93.816	

^a^ Stage1 as the reference.

^b^ Forward stepwise selection was conducted to select the model with minimal Akaike Information Criterion (AIC)

To find independent factors that may be associated with reporting through clinical surveillance, multivariate logistic regression was applied to analyze the correlation between dengue cases being clinically reported and relevant factors, after simultaneously controlling important confounding variables (using age <25 years and the early epidemic stage as reference groups). Forward selection was conducted to select the model with the minimal Akaike Information Criterion (AIC). As epidemic stage and age were strong confounders, stage-specific, age-specific, and overall models were compared after grouping clinical presentations of the same organ system and excluding independent variables with strong correlations. An adjusted odds ratio (aOR) and 95% confidence interval (CI) were calculated, with p < 0.05 considered statistically significant.

### Ethics

This study was approved by the National Taiwan University Hospital Institutional Review Board (IRB) (Approval Number: 200903086R). The process of reporting also followed government regulations for public health disease surveillance in Taiwan. Our data were fully de-identified and anonymized to protect privacy, and only retrospective aggregated data were used for further analyses and statistical tests.

## Results

### Overview of 2009–2010 Epidemic of Dengue

From July 11, 2009 to February 13, 2010, 632 (98.14%, 632/644) indigenous and 12 imported confirmed dengue cases were identified in Kaohsiung City. The predominant serotype for indigenous cases was DENV-3 (99.66%, 295/296). Among these DENV-3 cases, nine were diagnosed as DHF cases. Three of these nine DHF cases were fatal, with an overall case fatality rate of 0.5% (3/632).

### The Sensitivity of Clinical Surveillance of Dengue Cases

Among 632 confirmed cases in Kaohsiung, 96.4% were symptomatic, and 99.0% of the 609 symptomatic cases had sought medical care. Furthermore, only 86.4% of 603 symptomatic cases with medical visits were clinically diagnosed as dengue and reported to the KH-CDH. Considering the undetected cases throughout the epidemic, the estimated maximum sensitivity of this clinical surveillance through physician reporting was 82.4% (521/632). Of total Taiwan-CDC confirmed dengue cases, 521 (82.4%) and 111 (17.6%) cases were obtained from clinical and serological surveillance systems, respectively (Fig 1). Among 111 serologically detected cases through case-finding, 88 (79.3%) were symptomatic (20.7% asymptomatic).

### Temporal Characteristics of the Epidemic

This epidemic involved two waves peaking at the 32^nd^ and 46^th^ weeks and most DHF cases (88.9%, 8/9) occurred in the peaking period of the second wave (Fig 2A). The percentage of cases identified as dengue at the first medical visit was quite low (lightest blue in Fig 2B) during all three epidemic stages (16.3%, 15.9%, 12.7% for early, middle and late stages, respectively), and particularly lower in the 31^st^ and 43^rd^ weeks—before or at the beginning of each wave. Furthermore, over 80% of patients in the epidemic area required two or more clinical visits to be clinically diagnosed with dengue.

Serological surveillance picked up most dengue cases at the early stage, significantly higher than during the middle and late stages [30.4% (35/115), 13.7% (54/393), 17.7% (22/124), p = 0.0002] (Fig 2A). Interestingly, the number of medical visits and “detection delay days” increased particularly at the beginning period (31^st^–41^st^ weeks) and the time when dengue numbers climbed strikingly (42^nd^ to 43^rd^ weeks), with longer “detection-delay days” (exceeding 20 or 30 days). As the epidemic evolved from the 43^rd^ to 46^th^ week (climbing towards the peak), the weekly “detection-delay days” over three days were still quite high [64% (25/39), 75% (36/48), 58% (31/53), and 72% (49/68), respectively] and were parallel with high frequencies of over two medical visits [62%(24/39), 48% (23/48), 36% (19/53), and 34% (23/68), respectively] (Fig 2B and 2C). Therefore, higher detection-delay days and higher frequencies of hospital visits at more than two different medical institutions (hospital shopping) without confirmation might facilitate the virus spreading unnoticed. At the highest peak (46^th^ week), about 34% of cases were still identified only after three or more medical visits. Fig 2C shows that the weekly pattern of reported dengue cases through two surveillance systems paralleled the weekly confirmed cases. The means and medians of “detection-delay days” were around five days at the three stages without statistical differences but with differences in distributions. Particularly, in the two earliest weeks—the 31^st^ and 32^nd^ weeks—around 40% (2/5) and 18% (4/22) of dengue cases were reported longer than 7 days after the onset date (Fig 2C), which is also well correlated with higher hospital shopping (Fig 2B), implying that under lower alertness of physician in the initial epidemic stage, it would be more difficult to diagnose dengue patients who present atypical symptoms.

Moreover, the importance of early detection of dengue cases by both clinical and serological surveillance for increasing the effectiveness of dengue surveillance and early control is illustrated by spatio-temporal analysis of dengue cases identified by the two different surveillance approaches (S2–S4 Figs).

### Univariate Analyses between Clinically Reported and Unreported Dengue Cases

Table 1 summarizes all factors associated with the reported and unreported dengue cases through these two surveillance systems (Fig 1). First, the distributions of the visited clinics and hospital levels between these two groups of patients were significantly different (p = 0.0001). Other important factors included the number of medical visits, age, epidemic stages, and clinical symptoms/signs. Unreported dengue cases had (1) a higher percentage identified during the early epidemic stage (26.8% vs 15.4%, p = 0.02), (2) fewer patients seeking medical care (mean±S.D: 2.11±0.98 vs 2.50±1.00, p <0.01), (3) a lower percentage visiting district/regional hospitals or medical centers (56.1% vs 93.8%, p<0.01) before being reported, (4) younger age [0–25 years (45.5%, 10/22), 26–55 years (45.5%, 10/22), ≥56 years (9.0%, 2/22), p = 0.02], (5) a lower percentage of fever (82.9% vs 96.2%, p<0.01) but higher percentages of rash (64.4% vs 48.8%, p<0.01), itching (40.2% vs 19.6%, p<0.01), and diarrhea (34.1% vs 24.0%, p<0.05), and (6) longer “detection-delay days” (mean±S.D.: 8.7±7.2 vs 4.4 ±2.1, p<0.01) (Table 1).

To monitor surveillance dynamics, we investigated possible factors involved from early to middle and late epidemic stages (Table 2). Among clinically reported cases, a greater percentage in the late stage visited medical centers [31.4% vs 16.7%, p<0.01], although consistently high percentages (45.5%, 40.5% and 50%) of unreported cases came from local clinics through all three epidemic stages. Interestingly, over 90% of these unreported cases in the early stage made under three medical visits, with a significantly higher percentage than reported dengue cases in the early stage (p = 0.01). In other words, levels and frequencies of medical visits changed over epidemic stages. Particularly, younger patients and those presenting with less fever in the early stage or with diarrhea in the middle stage were more likely to be unreported. By the time the epidemic reached the middle and late stages, the detection-delay days of unreported cases were significantly longer (> 7 days: 69.0%, p<0.01; >7 days: 55.6%, p = 0.04, respectively).

### Multivariate Analyses between Clinically Reported and Unreported Dengue Cases

To identify important factors associated with clinical reporting after controlling for confounders, we applied forward stepwise regression models, including a full model with potential covariates and stage- or age-specific models (Tables 3 and 4). The full model (Table 3) shows that four factors were significantly associated with clinical reporting, including visiting hospitals or medical centers (aOR, 11.571; p < 0.001), middle epidemic stage (aOR, 2.404; p = 0.009), fever (aOR, 4.836; p < 0.001), and myalgia or arthralgia (aOR, 1.852; p = 0.034), whereas diarrhea (aOR, 0.468; p = 0.013) and rash or itching (aOR, 0.466; p = 0.010) were significantly associated with clinical under-reporting.

The three epidemic stage-specific models (Table 3) demonstrated that a higher frequency of medical visits and fever were positively associated with reporting, especially in the early stage, whereas cutaneous presentations (rash or itching) were negatively associated with reporting in the early and middle stages. In addition, visits to hospitals/medical centers were a significant factor for reporting throughout the three stages.

Since there was strong co-linearity between age and levels of medical institutes visited, we further examined three age-specific models (Table 4). The results show that different age groups (≤25, 26–55, ≥56 years) had different symptoms/signs that were positively or negatively associated with reporting dengue. The dengue cases with fever were more likely to be reported for the middle- and older-age groups, whereas myalgia (or arthralgia) and anorexia (or nausea or vomiting) were positively associated with dengue-reporting for younger and older age groups, respectively. However, diarrhea and rash (or itching) were negatively associated with dengue reporting in middle-age group patients.

## Discussion

Rapid detection of dengue cases for early public health intervention is crucial in areas where dengue is not endemic. While an unexpected increase in dengue cases still occurred in Kaohsiung of Taiwan in September 2009 after intensive surveillance effort in June and July, our results illustrate the problems of relying solely on clinical surveillance. As reporting of dengue cases required fever (ear temperature > 38°C) but not gastro-intestinal symptoms, continuous viral spread in communities was facilitated by both asymptomatic infection and atypical symptom presentations that were not picked up by front-line physicians in local clinics. Moreover, the addition of epidemiological characteristics was helpful in finding these cases and identifying various factors in surveillance from the early to middle and late epidemic stages that would otherwise have been missed if only the clinical surveillance had been used. Especially, frequency of medical visits and age were important in the early stage whereas patients’ having fever was influential for both early and late stages, and cases with rash/itching were more likely to be under-reported in the middle stage. Therefore, undetected sporadic cases at the early stage of an epidemic may lead to large-scale outbreaks if interventions are not immediately implemented [1, 23]. A domestic epidemic in a non-endemic country underscores the need for proactive dengue awareness, particularly in the time period before the epidemic season or during the initial days of an epidemic. Early detection of mild or asymptomatic dengue cases in these areas at this crucially important early time facilitates appropriate allocation of resources for intervention and halting further spread, thus reducing the chance of subsequent severe cases [9, 24].

In this study, active serological surveillance detected 30% (35/115) of cases in the early epidemic stage, showing that this approach is useful in detecting unreported, clinically atypical cases, and thus can complement passive clinical reporting [9]. Furthermore, asymptomatic individuals can also be reservoirs for the virus and may contribute to sustained viral transmission, possibly lengthening epidemic waves, particularly in areas with intense transmission rates (as observed in clusters of asymptomatic and symptomatic dengue cases) [25]. In fact, the ratio of asymptomatic to symptomatic dengue cases (detected by serological tests) is highly variable with respect to age [25, 26] and other factors, ranging from 1.1:1 to 13.1:1. This study demonstrates that serological surveillance is very important in the beginning of each wave when identification of cases at the first medical visit is quite low, or when hospital shopping is high or when detection-delay days are high before final confirmation of dengue. However, serological surveillance is neither cost-effective nor practical after the epidemic has peaked or after dengue cases have become wide spread. Public health intervention (e.g. cleaning breeding sites and killing mosquitoes) should then be the first priority. Delayed notification may lead to additional epidemic waves [27], highlighting the crucial role of serological surveillance in identifying asymptomatic or atypical dengue cases in a community, particularly for cases at the early stage of epidemics in non-endemic dengue areas.

Fever, the most sensitive symptom in clinical surveillance [28, 29], was presented in 90.3% (571/632) of dengue cases in this study. Interestingly, children infected with DENV generally showed milder symptoms and often did not have the classic “break-bone fever”. Percentages of arthralgia and myalgia were much lower in children than in adults in Taiwan [14], Thailand [30], Vietnam and Nicaragua [31]. In this Kaohsiung epidemic, older patients were more likely to be afebrile [0–25 y/o: 4.4% (5/113), 26–55 y/o: 7.2% (22/305), ≥56 y/o: 15.9% (34/214), p = 0.00073, by Fisher’s exact test]. It is noteworthy that, if passive surveillance was the only channel to detect cases, these afebrile or even asymptomatic dengue cases [9.7% (61/632) and 3.6% (23/632) identified from this study, respectively] could easily have been neglected [with a ratio of asymptomatic/symptomatic of 1/26.5 (23/609)]. In fact, 67.2% (41/61) of afebrile cases were detected through serological surveillance, which again illustrates the public health significance of other surveillance systems in addition to clinical reporting. On the other hand, serological surveillance is particularly useful in case-finding at the early stage of an epidemic. This happens when physicians are less alert due to low incidence, when patients present with non-specific clinical symptoms/signs, when children have high asymptomatic ratios, or when physicians were requested by local residents not to report them, so as to avoid insecticide spraying in their homes. Past experiences in Kaohsiung showed that early identification of the initial dengue cases through rapid implementation of integrated surveillance systems must be given the highest public health priority, followed closely by effective mosquito control measures. Both steps are crucial in restricting outbreaks to their sites of origin early [32] and thus avoiding sharp increases in severe dengue cases later on. This unique effort also explains why Taiwan prior to 2009, unlike other South East Asian countries [8], was able to prevent large numbers of severe dengue cases, which was largely due to the shortening of the duration per epidemic wave and minimizing transmission intensity among dengue clusters [24].

The factors leading to under-reporting of dengue cases through the clinical surveillance identified here include patients presenting with atypical or mild symptoms (e.g. no fever or diarrhea) who visited local clinics that were unaware of atypical presentations. Moreover, a younger population and either an overall lower frequency of seeking medical visits or visiting multiple medical facilities among those that did seek clinical support were often observed early in an epidemic. This is the first documentation showing that hospital shopping in Taiwan does influence the effectiveness of surveillance. It is very likely that higher frequencies of mild, non-specific presentations of dengue immediately after illness onset, ignorance of dengue’s broad spectrum of clinical manifestations, and the lack of awareness of risk factors (e.g. travel history to dengue-endemic/outbreak areas) may lead infected individuals to neglect seeking medical attention [33]. Consequently, physicians with low awareness of public health notifications [34] and those working in private clinics during the “high-risk” period were found to have reported fewer cases. This emphasizes the need to increase targeted communication to physicians in private clinics, raising overall clinical alertness and thus improving early detection of dengue at the community levels [33, 35]. While Kaohsiung has not yet become a dengue-endemic as of 2009, identifying all factors resulting in under-reporting, using data supported by surveillance, molecular epidemiological and seroepidemiological studies [4, 5, 17, 18] all together will make policy-makers to be better prepared for future outbreaks.

This study has four major limitations. First, recall bias may exist for early symptoms/signs and illness onset date. However, this bias should have little impact on our results because those with long “detection-delay days” (>14 days) accounted for only 2.4% (12/632) of confirmed cases. Secondly, clinical presentations were obtained from epidemiologists’ investigation through patients’ reporting and/or medical records, but the progression of disease and the differential diagnosis made at each medical visit of non-reported cases was unclear. Thirdly, the sensitivity of clinical surveillance (82.4%) could be an over-estimation because serological surveillance was conducted among only family members of confirmed cases who voluntarily gave blood (through contact tracing) but not all residents that lived within a 50- to 100-meter radius as was past practice. Lastly, the sensitivity of serological surveillance in the middle and late epidemic stages was under-estimated because this measure became a lower priority compared to removing mosquito breeding sites and killing mosquitoes.

Timely and effective risk communication with local health-care workers using up-to-date information (e.g. numbers of DF and severe dengue cases, case fatality rates, DENV serotypes, etc.), particularly with physicians working in private clinics, is vital to raise awareness of a potential epidemic. Most importantly, we advocate here that global dengue surveillance must not focus entirely on detecting DHF or severe dengue cases with warning signs as many dengue-endemic countries have done for the past, because severe and fatal cases generally occur more frequently during peak and later stages of an epidemic [33]. In other words, a new milestone of global collaboration is urgently needed to put more emphasis on better proactive surveillance from severe to mild and asymptomatic cases as well as even towards mosquito and environmental surveillance systems. Therefore, more buffer time is allowed to successfully contain small dengue clusters much earlier before large-scale outbreaks occur. The outbreaks of dengue in Japan in 2014 [7] and in several European countries in recent years suggest that, under the context of climate change and inadequate surveillance, there is a growing threat of dengue. Future efforts in minimizing the scale as well as the severity of dengue epidemics should include: (1) developing a web-based syndromic surveillance system for detecting early cases with dengue-like illness, using confirmed dengue cases to identify the earliest age-specific syndromic groups with higher sensitivity and specificity; (2) increasing sensitivity of clinical surveillance through reinforcing education for health care workers in local clinics and district hospitals by teaching physicians to find case early, record all symptoms/signs of patients, particularly in their early days of onset of dengue-like illness, and consider abnormal clinical manifestations in the elderly with co-morbidities; (3) implementing “integrated surveillance” that includes serological, entomological, and other proactive surveillance approaches to detect cases early in areas with high mosquito indices or breeding sites; (4) identifying temporal and spatial risks through timely spatial analyses of cases and clusters [10], and (5) establishing spatio-temporal multivariate prediction models involving meteorological, entomological, and environmental variables related to mosquito breeding sites and population movement patterns [36], and numbers of local dengue cases in previous time intervals [5]. Until an effective dengue vaccine becomes available [37], these endeavors plus up-to-date information on dengue cases in endemic countries from which most dengue cases are imported can strengthen the public’s and physicians’ risk awareness about dengue and thus minimize this health threat.

## Supporting Information

S1 Fig Location of Kaohsiung City (study area) in southern Taiwan. Most severe dengue cases occur in southern Taiwan, where mosquitoes of *Aedes aegypti* are distributed (i.e. below dotted line of Tropic of Cancer (23°27’N). Red polygon on map indicates location of Kaohsiung City (study area) in southern Taiwan. Brown line represents the Tropic of Cancer.
(EPS)

S2 Fig Three levels of population density in Kaohsiung in 2009. Spatial analyses showed dengue cases started mainly from Hsiao-Kang District closer to the harbor where foreign laborers from Southeast Asia live, rather than those with high population densities. Population density data in 2009 in Kaohsiung were obtained from the socio- economic database maintained by the Ministry of Interior, Taiwan [http://210.65.89.57/stat/web/portal/stat_portalhome.aspx].
(EPS)

S3 Fig The total laboratory confirmed dengue cases from early to middle and late stages of the epidemic in Kaohsiung, 2009–2010. The dengue cases in the early stage of the epidemic spread very fast and widely to the middle stage with overlapped dengue epidemic areas between the middle and late stages, indicating failure to contain the epidemic in the early stage. Weekly or biweekly total numbers of dengue cases (from both physicians’ reporting clinical surveillance and public health professionals’ case-finding effort in serological surveillance) in the early epidemic stage were further plotted to compare cases reported by physicians through clinical surveillance versus those identified from case-finding by public health professionals through serological surveillance (S2(C) Fig). We generated the maps by ArcGIS 10.2 (ESRI Inc., Redlands, CA, USA). The world base map was produced from a publicly available administrative boundary map maintained by the DIVA-GIS website (http://www.diva-gis.org/Data), and Taiwan’s village map was also obtained from a publicly available map maintained by the Ministry of Interior, Taiwan (http://data.gov.tw/node/7440).
(EPS)

S4 Fig Spatio-temporal analyses of Li-specific confirmed dengue cases detected through the two surveillance systems in Kaohsiung, 2009–2010. The weekly or biweekly numbers of confirmed dengue cases reported through clinical surveillance versus detected by case-finding through serological surveillance are shown as pie charts by cases’ residential “li” (the basic neighborhood-level administration unit in Taiwan). It was found that the dengue cases increased quickly at the early stage, from the 31^st^ to the 33^rd^ weeks when serological surveillance (marked in light green) finally detected cases with atypical symptoms/signs. In addition, the cases had already spread during the 2^nd^ week of the outbreak, and serological surveillance focusing only on family contacts might be inefficient in finding cases in the first four weeks (31^st^ to 34^th^ week). Two sources of the dengue cases were from case finding through serological surveillance by public health professionals versus reported by physicians through clinical surveillance during initial 10 weeks (weeks 31–40, 2009) at early epidemic stage in Kaohsiung. Li-specific percentages of laboratory-confirmed dengue cases detected through clinical surveillance (in magenta) or serological surveillance (in light green) are shown by pie charts to protect privacy.
(EPS)
