# Correction: The Critical Role of Early Dengue Surveillance and Limitations of Clinical Reporting - Implications for Non-Endemic Countries

**DOI:** 10.1371/journal.pone.0201502

**Published:** 2018-07-24

**Authors:** Jui-Hung Kao, Chaur-Dong Chen, Zheng-Rong Tiger Li, Ta-Chien Chan, Tsung-Hwa Tung, Ying-Hsia Chu, Hau-Yuan Cheng, Jien-Wei Liu, Fuh-Yuan Shih, Pei-Yun Shu, Chien-Chou Lin, Wu-Hsiung Tsai, Chia-Chi Ku, Chi-Kung Ho, Chwan-Chuen King

The sixth author’s name is spelled incorrectly. The correct name is: Ying-Hsia Chu.
